# Decreased grip strength, muscle pain, and atrophy occur in rats following long‐term exposure to excessive repetitive motion

**DOI:** 10.1002/2211-5463.12315

**Published:** 2017-10-16

**Authors:** Mitsuhiro Fujiwara, Masahiro Iwata, Takayuki Inoue, Yosuke Aizawa, Natsumi Yoshito, Kazuhiro Hayashi, Shigeyuki Suzuki

**Affiliations:** ^1^ Program in Physical and Occupational Therapy Nagoya University Graduate School of Medicine Japan; ^2^ Department of Rehabilitation Kamiiida Rehabilitation Hospital Nagoya Japan; ^3^ Department of Rehabilitation Faculty of Health Sciences Nihon Fukushi University Handa Aichi Japan; ^4^ Department of Rehabilitation Nagoya University Hospital Japan; ^5^ Department of Rehabilitation Japanese Red Cross Nagoya Daiichi Hospital Nagoya Japan; ^6^ Department of Rehabilitation Nagoya City University Hospital Japan; ^7^ Multidisciplinary Pain Center Aichi Medical University Nagakute Aichi Japan

**Keywords:** autophagy‐lysosome system, grip strength, muscle atrophy, muscle pain, ubiquitin‐proteasome system, work‐related musculoskeletal disorders

## Abstract

Work‐related musculoskeletal disorders (WMSD) are caused by the overuse of muscles in the workplace. Performing repetitive tasks is a primary risk factor for the development of WMSD. Many workers in highly repetitive jobs exhibit muscle pain and decline in handgrip strength, yet the mechanisms underlying these dysfunctions are poorly understood. In our study, rats performed voluntary repetitive reaching and grasping tasks (Task group), while Control group rats did not perform these activities. In the Task group, grip strength and forearm flexor withdrawal threshold declined significantly from week 2 to week 6, compared with these values at week 0 (*P *<* *0.05). Relative muscle weight and muscle fiber cross‐sectional area of flexor digitorum superficialis (FDS) muscles decreased significantly in the Task group, compared with the Control group, at 6 weeks (*P *<* *0.05 and *P *<* *0.01, respectively). Nerve growth factor, glial cell line‐derived neurotrophic factor, and tumor necrosis factor α‐expression in FDS muscles were not significantly different in Control and Task groups at 3 and 6 weeks. At 6 weeks, the Task group had elevated MuRF1 protein levels (*P *=* *0.065) and significant overexpression of the autophagy‐related (Atg) proteins, Beclin1 and Atg5–Atg12, compared with in the Control group (both *P *<* *0.05). These data suggested that long‐term exposure to excessive repetitive motion causes loss of grip strength, muscle pain, and skeletal muscle atrophy. Furthermore, this exposure may enhance protein degradation through both the ubiquitin‐proteasome and autophagy‐lysosome systems, thereby decreasing skeletal muscle mass.

**Abbreviations**
Atg: autophagy‐related
Atrogin‐1: muscle atrophy F‐box/Atrogin‐1
CBB: coomassie brilliant blue
CSA: cross‐sectional area
DOMS: delayed‐onset muscle soreness
EDL: extensor digitorum longus
FDS: flexor digitorum superficialis
GDNF: glial cell line‐derived neurotrophic factor
IL: interleukin
LC3: microtubule‐associated protein 1 light chain 3
MuRF1: muscle RING finger 1
NGF: nerve growth factor
PVDF: polyvinylidene difluoride
qRT‐PCR: quantitative reverse transcription PCR
SEM: standard error of the mean
TNF: tumor necrosis factor
WMSD: work‐related musculoskeletal disorders

Work‐related musculoskeletal disorders (WMSD) are defined as injuries or musculoskeletal effects caused by exposure to certain risk factors in the workplace. Risk factors for WMSD include repetition, force, and awkward or static postures. Previous research showed that in highly repetitive jobs (a cycle time < 30 s or more than 50% of the time performing same type of fundamental cycle), there was a 2.8 odds ratio of injury compared with in low repetition jobs 1. Of female workers in highly repetitive jobs, 23% reported muscle pain in forearm and hand muscles 1. Furthermore, women seeking medical care for WMSD of the upper extremities also showed decreased handgrip strength, and these symptoms were related to their levels of perceived physical exertion 2.

Using rat models, previous studies have investigated the pathophysiology of injuries of the upper forelimb caused by voluntary repetitive reaching and grasping tasks 3, 4. Several investigators reported that the repetitive tasks caused decreased grip strength 5, 6, 7, 8, 9. Barbe *et al*. 5 reported that performance of voluntary repetitive reaching and grasping tasks led to increased levels of tumor necrosis factor (TNF)‐α, interleukin (IL)‐1α, and IL‐1β in rat forearm flexor muscles. Furthermore, this task led to elevated levels of substance P and its preferred receptor, neurokinin‐1, in the spinal cord dorsal horn 10. In addition, previous studies showed that high repetition tasks increased fibrogenic‐related proteins in skeletal muscle or tendon 7, 8, 9. Several inflammatory cytokines, including TNF‐α, IL‐1α, IL‐1β, were implicated in pain. Substance P is another major mediator of neuropathic inflammatory pain. Therefore, muscle pain would be expected to occur in volunteers performing repetitive reaching and grasping tasks. Recently, Xin *et al*. 11 studied skin pain, often assessed with a von Frey hair test, in a WMSD model. However, according to Nasu *et al*. 12, it is necessary to use a probe with a diameter of at least 2.6 mm to evaluate the mechanical threshold of skeletal muscle. Thus, while factors affecting decreased grip strength after repetitive reaching and grasping tasks have been proposed in several studies 5, 6, 7, 8, 9, those affecting pain in such models have not been identified. It remains to be demonstrated that a rat model for repetitive tasks exhibits similar muscle pain as that seen in workers with highly repetitive jobs.

In several other animal models, muscle pain was produced by exercise stresses different from the repetitive reaching and grasping tasks. Some animals were forced to exercise by repetition of eccentric contractions. After such exercise, they experienced delayed‐onset muscle soreness (DOMS) and declines in mechanical withdrawal threshold in the muscle 13, 14. In a previous study, physical exercise, including eccentric contractions, induced expression of nerve growth factor (NGF), glial cell line‐derived neurotrophic factor (GDNF), and TNF‐α in skeletal muscle and, consequently, led to decreased muscle mechanical thresholds 13, 15, 16. NGF, produced in skeletal muscle after ischemia 17 or nerve injury, can sensitize muscle nociceptors 18. Like NGF, GDNF is also responsible for growth and maintenance of certain neurons. In rats, injection of either NGF or GDNF into skeletal muscle led to a decline in the mechanical threshold 16, 19. Therefore, we hypothesized that performance of repetitive reaching and grasping tasks by rats would lead to upregulation of NGF, GDNF, and TNF‐α and to declines in mechanical thresholds in muscle.

It is further worth noting that generally, pain reduces muscle strength 20, 21, 22. However, studies showed strong correlations between muscle force and either muscle volume or cross‐sectional area (CSA) 23, 24, 25. Hence, not only muscle pain, but also skeletal muscle atrophy, may be responsible for the decline in grip strength caused by repetitive reaching and grasping tasks. Changes in skeletal muscle mass, in rats performing the tasks, have not been investigated.

Skeletal muscle adaptation depends on dynamic interplay between changes in muscle protein synthesis and degradation. Both the ubiquitin‐proteasome and autophagy‐lysosome systems are involved in the degradation of skeletal muscle proteins.

The ubiquitin‐proteasome system degrades soluble and myofibrillar proteins 26. Specificity of substrate tagging is ensured by ubiquitin ligases. For example, levels of two muscle‐specific ligases, muscle RING finger 1 (MuRF1) and muscle atrophy F‐box/Atrogin‐1 (Atrogin‐1), were increased in several instances of catabolism 27. MuRF1 and Atrogin‐1 are believed to selectively bind substrates for ubiquitination and subsequent degradation by the 26S proteasome. Thus, increased MuRF1 and Atrogin‐1 expression following an atrophy‐inducing stressor is attributed to the shift in protein balance from net synthesis to net degradation 28. The process of autophagy begins with autophagosome formation, in which a double membrane is formed around a portion of the cytoplasm containing proteins and/or organelles. Autophagosome formation is under the control of proteins encoded by autophagy‐related (Atg) genes. In particular, two ubiquitin‐like conjugation systems were described. In the first, the Atg5–Atg12 complex can interact with Atg16 to participate in autophagosome membrane formation. The second involves Atg8, also known in mammals as microtubule‐associated protein 1 light chain 3 (LC3). LC3II was implicated in substrate selection for degradation and in membrane fusion and, when bound to phospholipid, is known as LC3II 29. Among proteins implicated in control of the autophagy process, the Atg6 homolog Beclin1 appears to be central for the initiation of sequestration 30, 31.

In one report, long‐term running exercise led to increased MuRF1 protein expression and proteasome activity 32 and increased levels of Atg proteins Beclin1 and LC3II, suggesting increased basal autophagy 33, in plantaris muscle of mice. Thus, long‐term running exercise is believed to promote muscle protein degradation through these two systems. However, changes, during repetitive reaching and grasping tasks, in molecules involved in muscle protein degradation were not yet investigated.

Therefore, the first purpose of our study was to investigate factors involved in decreased grip strength, from the perspective of muscle pain and skeletal muscle mass, associated with voluntary repetitive reaching and grasping tasks in the rat. The second purpose was to examine key regulators of muscle pain associated with the task. The third purpose was to evaluate repetitive task‐dependent activation of the ubiquitin‐proteasome and autophagy‐lysosome systems. In this study, we chose a rat model not involving use of force. As shown by Barbe *et al*. 5 and Clark *et al*. 6, rats performing continued repetitive reaching and grasping tasks (high repetition negligible force model) exhibited dysfunctions similar to those in humans. This indicated that high‐frequency motion, not necessarily involving high force, induces WMSD. Thus, in our study, we investigated the hypothesis that long‐term exposure to excessive repetitive motion would cause loss of grip strength, muscle pain, and skeletal muscle atrophy and, furthermore, that such atrophy would involve enhanced protein degradation through the ubiquitin‐proteasome and autophagy‐lysosome systems.

## Materials and methods

### Animals

All animal procedures were performed under the animal care guidelines of Nagoya University. A total of 41 female Sprague‐Dawley rats, aged 10 weeks, were used for this study. Rats were from Japan SLC (Hamamatsu, Japan) and were housed under controlled temperature (25 °C) and lighting (8:00–20:00 light) conditions. Rats were divided randomly into food‐restricted control (Control, *n* = 19) or task (Task, *n* = 22) groups. Control and Task groups were restricted from food for 5 or 8 weeks. These rats were food deprived so that they maintained 80%–90% of full body weight, as defined by the weights of age‐matched normally fed rats. Rats were weighed weekly and their food was adjusted accordingly 3, 4, 6. After a 2‐week training period (described below) and the first 3 weeks of the Task period (this time is known as ‘3 weeks’ in our study), the rats in each group were further subdivided to perform certain analyses. The reason was to avoid potential artifacts in the muscle tissue analyses introduced by prior behavioral testing. Thus, a subset of the Control group at 3 weeks (*n* = 6) and Task group at 3 weeks (*n* = 6) were never subjected to behavioral analysis. This enabled their muscle samples to be used for both histological (i.e., measurements of relative muscle weight and muscle fiber CSA) and molecular biological and biochemical (i.e., mRNA expression relevant to muscle pain, ubiquitin‐proteasomes, and autophagy‐lysosomes) analyses because these rats had no potential effects of behavioral analysis. For rats providing tissue at the 6‐week time point, the type of behavioral testing they had been subjected to determined which types of analyses could be performed on their tissues. Thus, behavioral analysis data (grip strength and pain) were examined only in certain rats and these were measured for the six consecutive weeks of the Task period (i.e., the Control group in which grip strength was measured (*n* = 6), the Control group in which muscle pain was measured (*n* = 7), the Task group in which grip strength was measured (*n* = 6), and the Task group in which muscle pain was measured (*n* = 10). Grip strength and pain were performed on separate groups of rats but, for each parameter, the same rats were compared each week. For the ‘week 6’ tissue samples, histological analysis (i.e., measurements of relative muscle weight and muscle fiber CSA) and molecular biological and biochemical analyses (i.e., mRNA and protein expression relevant to ubiquitin‐proteasomes and autophagy‐lysosomes) were performed using muscle tissue from rats in which grip strength had been measured. In contrast, NGF, GDNF, and TNF‐α levels were measured using muscle tissue from rats in which muscle pain had been measured. The reason for this was the concern that applying mechanical stimulation to the skeletal muscle, necessary to measure muscle pain, would affect skeletal muscle size. For example, in previous studies, skeletal muscle atrophy was suppressed by mechanical stimulation such as stretching 34, 35. Therefore, we did not want to analyze skeletal muscle size in rats that had been tested for pain. It should be noted that the body weights did not differ between the Control and Task groups throughout the experimental period. Thus, differences in muscle, such as fat content, caused by excessive weight would not have confounded our histological analyses.

### Voluntary repetitive reaching and grasping tasks

**Figure 1:**
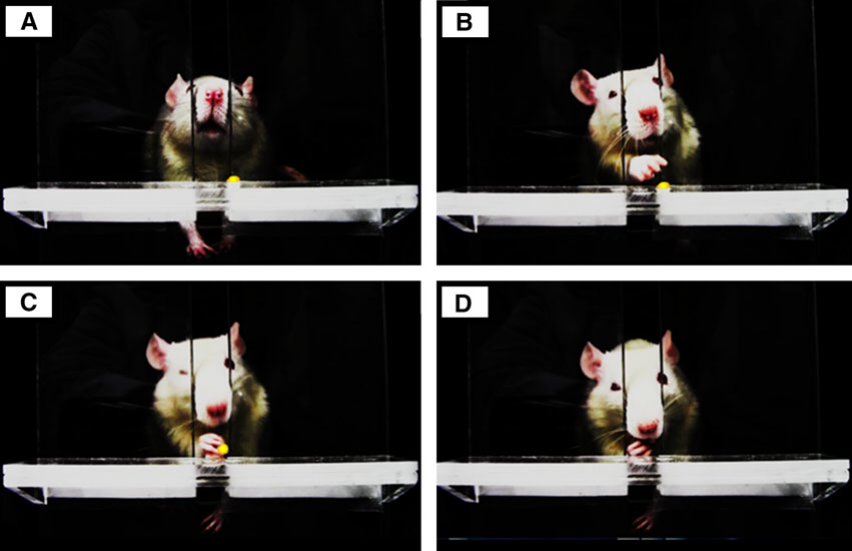
Voluntary repetitive reaching and grasping for food pellets. During the task, the rat repeatedly performs the action shown in the photograph once every 15 s (A–D). (A) The food pellet is dispensed on the shelf attached to the test box placing the rat. (B,C) The rat reaches and grasps for the food pellet placed on the shelf. (D) The rat eats the food pellet grasped in its paw.

Rats were placed in the test box, as described by Metz and Whishaw 36. There was a 1.3‐cm‐wide vertical opening in the middle of the front wall enabling rats to reach for pellets placed on a shelf. Food pellets (45 mg; BioServ, Frenchtown, NJ, USA) were dispensed every 15 s during the task. The rats performed the voluntary repetitive reaching and grasping tasks to obtain food pellets (Fig. 1). When a rat appeared likely to react to the pellets, an observer played a sound that was timed with pellet distribution 3, 4. In addition, the observer adjusted the position of the pellet, so that the rats could grip the pellet with only one paw at a time. Control and Task groups were initially trained for 2 weeks to learn the voluntary repetitive reaching and grasping task for obtaining a food pellet. After the initial training period, only the Task group rats performed the repetitive reaching and grasping tasks, for 2 h·day^−1^, 3 days per week, for 3 or 6 consecutive weeks. An observer counted the number of reaches and grasps, how many food pellets were obtained, and task durations. The task duration was defined as the total time that the rat participated in the task, and was as long as 2 h·day^−1^. These task parameters were used as the indicators of physical load. As previously described 3, this model used a ‘high repetition, negligible force’ task.

### Forelimb grip strength measurement

Rats were lifted by the tail and induced to grasp a rigid bar attached to a digital force gauge (Aikoh Engineering Corporation, Osaka, Japan). Each rat was gently pulled backward by the tail and the tension reading of the digital force gauge, just before the rat released the bar, was defined as grip strength. The test was performed five consecutive times and the highest value from the five trials was recorded as the grip strength.

### Withdrawal threshold measurement

**Figure 2:**
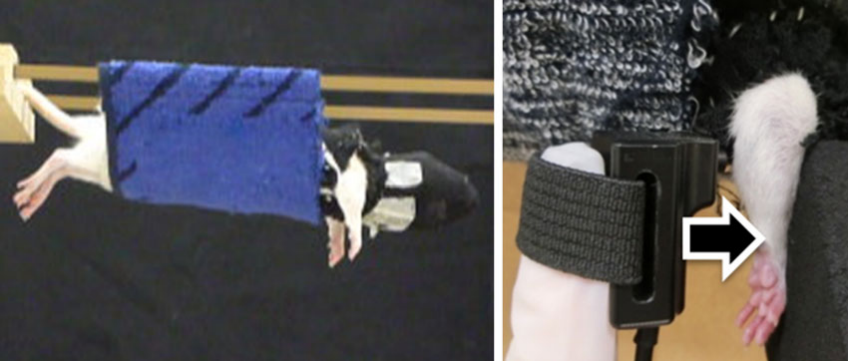
Mechanical hyperalgesia analysis measuring forearm withdrawal threshold. Two photographs illustrate the procedure, as described in detail in the Materials and methods. Each rat, with head and trunk covered, was suspended in a homemade hammock (left). This position allowed the forelimbs to freely move, with the arrow indicating the direction of mechanical forces applied to the forearm flexors (right).

Muscular mechanical hyperalgesia was measured as a forearm withdrawal threshold, in response to gradually increasing mechanical forces applied to the forearm flexors [flexor digitorum superficialis (FDS) muscle included]. The method was a modification of that described by Nakano *et al*. 37 (Fig. 2). Briefly, the head and trunk of the rat were wrapped with a cloth and the rat was suspended in a homemade hammock. We confirmed that, under these conditions, the forelimb position was freely movable. Forearm withdrawal threshold was quantified with a Pressure Application Measurement device (Ugo Basile, Comerio, Italy) equipped with a handmade round‐headed probe (5 mm tip diameter). It has been previously shown that a probe with a tip diameter of 5 mm allows measurements of the muscle mechanical nociceptive threshold 12. The pressure required to elicit forelimb withdrawal was determined, and measurements were taken 5 times at 1‐min intervals. The mean value of these measurements was used as the forearm flexor withdrawal threshold.

### Tissue collection

Tissues were collected from a total of 34 rats, from the Control group at 3 weeks (*n* = 6) and 6 weeks (*n* = 11) and from the Task group at 3 weeks (*n* = 6) and 6 weeks (*n* = 11). This tissue collection was performed 24–36 h after completion of the final task, to ensure that measurements reflected the chronic effects of long‐term exposure to repetitive motion, rather than immediate effects of the last exercise. Similarly, in a previous study 5, muscle tissue was collected 18–36 h after the final task. Rats were euthanized by pentobarbital overdose (Nembutal; 120 mg·kg^−1^). FDS muscle from each reaching limb was harvested immediately after euthanasia. After harvesting, each muscle sample was cut into two pieces that were preserved, respectively, for molecular biological and biochemical analyses and histological analysis. Specifically, muscles were cut across the midbelly portion and the distal part was used for molecular biological and biochemical analyses. The proximal part was used for histological analysis. The midbelly portion of the muscle was cut to measure the CSA at the site where the muscle fiber became thickest 38, 39. Muscle samples were flash‐frozen and stored at −80 °C until analyzed.

### Morphological analysis of FDS muscles

A total of 24 frozen muscle samples were analyzed, from the Control group at 3 weeks (*n* = 6) and 6 weeks (*n* = 6) and from the Task group at 3 weeks (*n* = 6) and 6 weeks (*n* = 6). These samples were embedded in optimal cutting temperature compound (Sakura Finetek, Torrance, CA, USA), and cross sections (8 μm) were cut with a cryostat (CM1510S; Leica, Wetzlar, Germany). These muscle cryosections were stained with hematoxylin and eosin (H&E), and images of the stained samples were obtained with an optical microscope (BZ‐9000; KEYENCE, Osaka, Japan). Muscle fiber CSAs were measured from captured images using image j software (National Institutes of Health, Bethesda, MD, USA). Analysis was performed on at least 200 muscle fibers per muscle, and these values were averaged.

### RNA isolation

Muscles were homogenized in ice‐cold Trizol Reagent (Invitrogen, Carlsbad, CA, USA) according to the manufacturer's instructions. After incubation for 5 min at room temperature, the mixture was centrifuged at 15 000 ***g*** for 10 min at 4 °C. The supernatant was transferred to a tube, a 1/5 volume of chloroform was added, and sample was mixed. The mixture was incubated for 5 min at room temperature, then centrifuged at 15 000 ***g*** for 10 min at 4 °C. The aqueous layer was transferred to another tube and mixed with 0.8 volume of isopropanol by pipetting. The mixture was incubated for 10 min at room temperature, then centrifuged at 15 000 ***g*** for 10 min at 4 °C. The RNA pellet was washed with 70% ethanol and centrifuged at 15 000 ***g*** for 10 min at 4 °C. RNA was dissolved in 20 μL RNase‐free water with RNase inhibitor (final concentration 2 μg·μL^−1^) and further subjected to DNase treatment and reverse transcription using a ReverTra Ace qPCR RT Master Mix (Toyobo, Osaka, Japan).

### Real‐time quantitative reverse transcription PCR

Quantitation of gene expression was determined by real‐time quantitative reverse transcription PCR (qRT‐PCR). qRT‐PCR mixture contained 2 μL cDNA, 10 μL TaqMan^®^ Fast Advanced Master Mix (Life Technologies, Carlsbad, CA, USA), and 1 μL primers. The PCR analysis was performed with a StepOnePlus Real‐Time PCR system (Applied Biosystems, Foster City, CA, USA), using the ΔΔCt method. The primers were NGF (Ngf, Rn01533872_m1), GDNF (Gdnf, Rn00569510_m1), MuRF1 (Trim63, Rn00590197_m1), Atrogin‐1 (Fbxo32, Rn00591730_m1), Beclin1 (Becn1, Rn00586976_m1), Atg5 (Atg5, Rn01767063_m1), LC3 (Anxa3, Rn00563181_m1), and β‐actin (Actb, Rn00667869_m1), all from TaqMan Gene Expression Assays (Life Technologies). β‐Actin was used as an endogenous control to normalize results for each sample.

### Protein isolation

Frozen muscle samples were homogenized in RIPA buffer plus 10% protease inhibitor cocktail (1 mg protein/10 ml RIPA), tissue lysates were centrifuged at 14 000 ***g*** for 10 min, and the supernatants were collected. Total protein was determined using the BCA‐200 protein assay kit (Pierce, Rockford, IL, USA).

### Western blot analysis

After measuring total protein content, each extract was adjusted to a concentration of 2 μg·μL^−1^ with an appropriate volume of RIPA buffer for SDS/PAGE. Lysates were mixed with equal volumes of EzApply (Atto, Tokyo, Japan) containing 100 mm Tris/HCl buffer (pH: 8.8), 2% SDS, 20% sucrose, 0.06% bromophenol blue, and 100 mm DTT and were heated at 95 °C for 5 min. For SDS/PAGE, 15–30 μL aliquots of samples were loaded into individual lanes and separated on a precast polyacrylamide gel (10%–18%; Bio‐Rad, Hercules, CA, USA) at a constant voltage of 200 V for 30–45 min. Protein bands were then transferred to a 0.2‐μm polyvinylidene difluoride (PVDF) membrane (Bio‐Rad) by electroblotting at a constant current of 1.3 A for 5 or 7 min, using a rapid transfer system (Trans‐Blot Turbo; Bio‐Rad). After transfer, western blotting was performed with a protein detection system (SNAP i.d. 2.0; Merck Millipore, Billerica, MA, USA). Blots were blocked against nonspecific reactions with 0.5% (w/v) nonfat‐dried milk or 1% (w/v) bovine serum albumin, diluted in Tris‐buffered saline containing either 0.1% (v/v) Tween 20 (TBS‐T), or with PVDF Blocking Reagent (Can Get Signal; Toyobo). Blots were then incubated overnight at 4 °C with antibodies directed against NGF (sc‐548; Santa Cruz Biotechnology, Santa Cruz, CA, USA), GDNF (sc‐328, Santa Cruz Biotechnology), MuRF1 (ab77577; Abcam, Cambridge, MA, USA), Beclin1 (PD017; MBL International Corporation, Nagoya, Japan), or Atg5–Atg12 (PM050; MBL International Corporation). Blots were then washed, incubated with the appropriate secondary antibodies, and washed again. Stained bands were then visualized using the ECL Prime Western Blotting Detection System (RPN2232; GE Healthcare Japan, Tokyo, Japan) according to the manufacturer's instructions. Images were captured and documented with a CCD system (Light Capture II; Atto). After measurements, membranes were stripped using stripping buffer (Restore Plus, Pierce, Rockford, IL, USA) and stained with coomassie brilliant blue (CBB) to verify equal loading in all lanes 40. Densitometric analysis of these images was performed using imaging analysis software (CS analyzer 3.0; Atto) and normalized to the optical density of the CBB stain of each band.

### ELISA

Tissue lysates from the Control group at 6 weeks (*n* = 5) and Task group at 6 weeks (*n* = 5) were analyzed using an ELISA kit for rat TNF‐α (Ray Biotech, Norcross, GA, USA), following the manufacturer's protocol. The minimum assay sensitivity for TNF‐α was 25 pg·mL^−1^. Data (pg protein) were normalized to μg total protein.

### Statistical analysis

Values are presented as means ± standard error of the mean (SEM). Comparison of body weights, grip strengths, and mechanical hyperalgesia values was made by two‐way repeated‐measures ANOVA, followed by Holm–Sidak multiple comparison tests. Other comparisons were made using Student's *t*‐test, for time‐matched data from two groups. For transparency, both significant differences (*P *<* *0.05) and trends (0.05 ≤ *P *<* *0.1) are reported, where appropriate. A *P* value of < 0.05 was considered significant. Data were analyzed using sigmastat 4.0 statistical software (Systat Software Inc., San Jose, CA, USA).

## Results

### Parameters from the voluntary repetitive reaching and grasping task

The rats performed ~ 4000 reaches and grasps during the first week and ~ 2000 additional reaches and grasps during each subsequent week (Table 1). As a result, the total number of reaches and grasps throughout the experimental period exceeded 14 000. There were more reaches and grasps during the first week, compared with other weeks, because the rats attempted the task even when pellets were not delivered. Beginning in the second week, rats attempted the reach only when pellets were distributed. In total, the rats obtained ~ 1100 food pellets per week. Thus, the total number of food pellets obtained reached ~ 6600 throughout the experimental period. The rats participated in the task for an average of ~ 5.6 h each week, or ~ 34 h throughout the 6‐week experiment.

**Table 1:** Parameters from voluntary repetitive reaching and grasping task

	1 week	2 weeks	3 weeks	4 weeks	5 weeks	6 weeks
Total reaches and grasps (*n*)	4019.0 ± 533.5	6682.2 ± 775.5	8909.0 ± 974.9	10904.2 ± 1161.1	12853.5 ± 1313.7	14758.3 ± 1459.7
Total food pellets obtained (*n*)	1047.7 ± 60.3	2117.8 ± 88.1	3267.0 ± 116.8	4355.0 ± 176.1	5457.5 ± 235.4	6579.2 ± 292.8
Total task duration (h)	5.6 ± 0.3	11.2 ± 0.5	17.0 ± 0.7	22.6 ± 1.0	28.2 ± 1.2	33.9 ± 1.3

Data are presented as mean ± SEM.

### Body weight and grip strength

**Figure 3:**
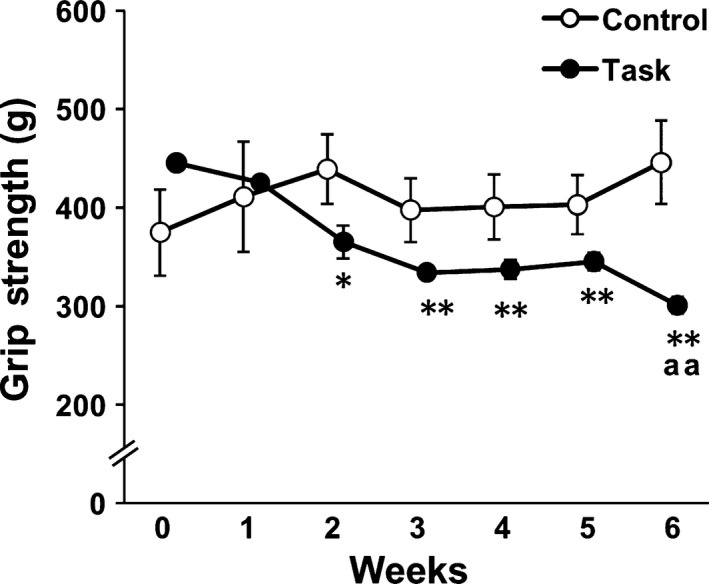
Changes in grip strength. Data are means ± SEM. *P <* *0.05, **P <* *0.01, compared with week 0. ^aa^ *P* < 0.01, compared with time‐matched rats from the Control group (*n* = 6 per group).

Body weights gradually increased in both Control and Task groups from week 0 (204.7 ± 2.7 and 215.3 ± 2.4 g, respectively) to week 6 (231.7 ± 6.1 and 237.5 ± 3.0 g, respectively). There were no significant differences in body weight between Control and Task groups. In the Control group, grip strengths did not change, compared with those at week 0, throughout the experimental period (Fig. 3). In the Task group, grip strengths declined significantly from week 2 to week 6, compared with at week 0 (*P *<* *0.05), and were significantly lower than in the Control group at 6 weeks (*P *<* *0.01). Grip strengths of the Task group decreased rapidly until week 3 and maintained an ~ 25% decrease, compared with initial values, from week 3 to week 5. The maximum decrease in grip strength in the Task group was evident at 6 weeks, a 32.4% decrease.

### Forearm flexor withdrawal threshold

**Figure 4:**
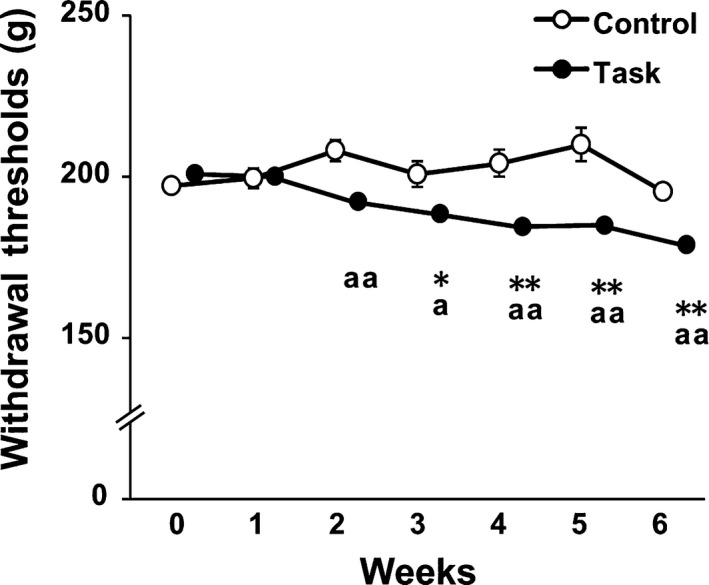
Change in forearm flexor withdrawal thresholds. Data are means ± SEM. *P <* *0.05, **P <* *0.01, compared with week 0. ^a^ *P* < 0.05, ^aa^ *P* < 0.01 compared with time‐matched rats from Control group (*n* = 7 in Control group and *n* = 10 in Task group).

Muscle mechanical hyperalgesia was measured as forearm flexor withdrawal threshold values. In the Control group, forearm flexor withdrawal thresholds did not change, compared with those at week 0 (Fig. 4). In the Task group, forearm flexor withdrawal thresholds declined significantly from week 3 to week 6, compared with initial values (*P *<* *0.05). These values were significantly lower than those in the Control group from week 2 to week 6 (*P *<* *0.05). Forearm flexor withdrawal threshold values for rats in the Task group were gradually decreased up to 6 weeks. The maximum decrease in forearm flexor withdrawal threshold in the Task group was that measured at 6 weeks, a decrease of 11.0%, compared with initial values.

### Morphological analysis of FDS muscle

**Figure 5:**
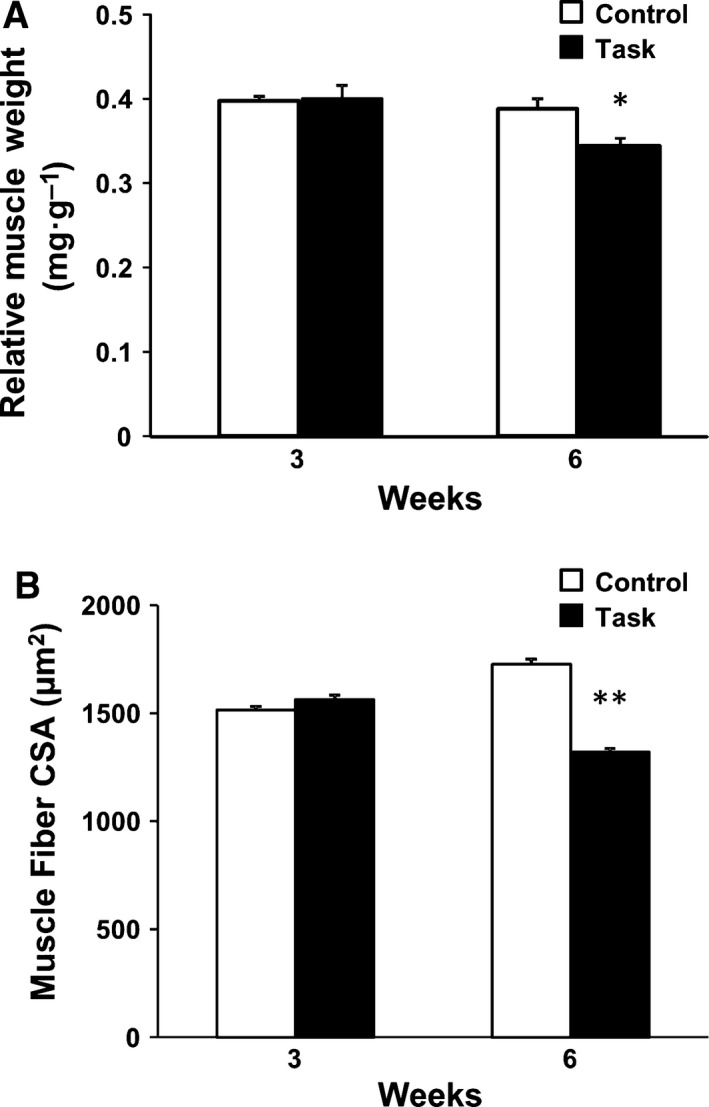
Morphological changes in FDS muscles at 3 and 6 weeks. (A) Relative muscle weight, (B) muscle fiber CSA. Data are means ± SEM. *P <* *0.05, **P <* *0.01, compared with time‐matched rats from Control group (*n* = 6 per group).

Relative muscle weight (ratio of muscle wet weight normalized to body weight) and muscle fiber CSA values of FDS were not significantly different between the two groups at 3 weeks (Fig. 5A,B). However, both values were significantly lower in the Task group than in the Control group at 6 weeks (*P *<* *0.05 and *P *<* *0.01, respectively).

### NGF, GDNF, and TNF‐α expression in FDS muscle

**Figure 6:**
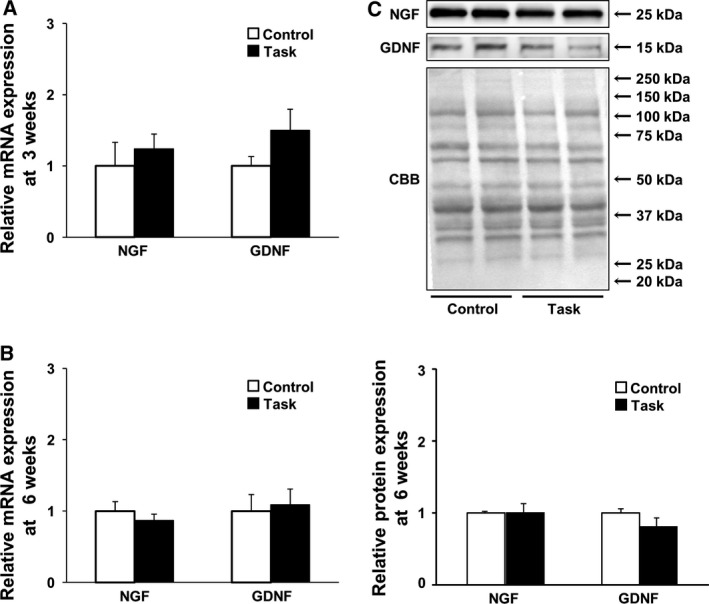
Expression of neurotrophic factors in FDS muscles. (A) NGF and GDNF mRNA expression at 3 weeks (*n* = 6 per group); (B) NGF and GDNF mRNA expression at 6 weeks (*n* = 5 per group); (C) NGF and GDNF protein expression at 6 weeks. Representative blots depicting NGF and GDNF are shown. Quantitative analysis is shown in the lower panel. Results are reported as fold changes with respect to control levels, which were arbitrarily set to 1. Data are means ± SEM.

**Figure 7:**
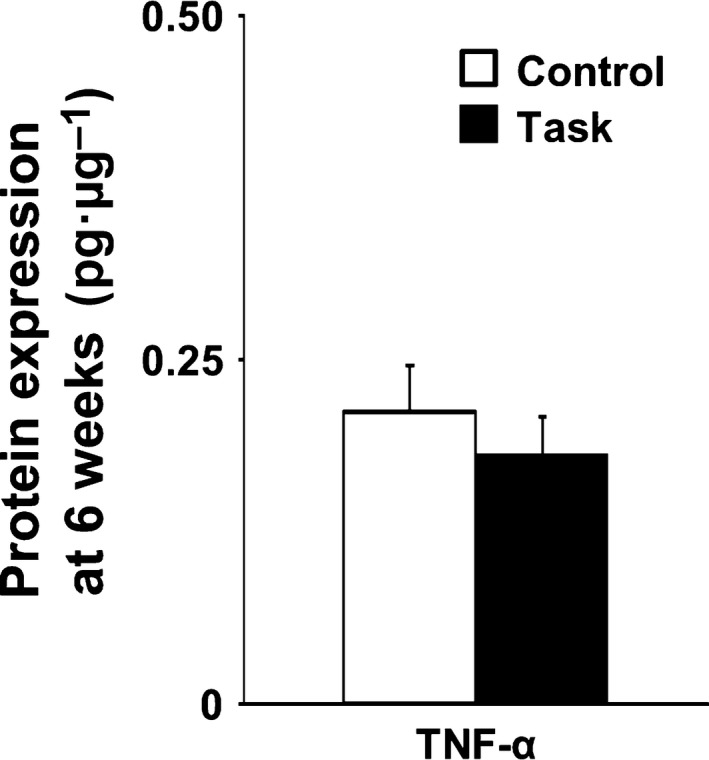
Expression of TNF‐α protein in FDS muscles at 6 weeks. Data are means ± SEM (*n* = 5 per group).

The neurotrophic factors NGF and GDNF and the inflammatory cytokine TNF‐α have been implicated in muscle mechanical hyperalgesia 16, 19, 41, 42. We found no significant differences, between Control and Task groups, in NGF or GDNF mRNA levels at 3 and 6 weeks (Fig. 6A,B). Consistent with this, western blotting showed no differences in NGF and GDNF protein levels in the two groups at 6 weeks (Fig. 6C). There were also no differences in TNF‐α levels, determined by ELISA, in the two groups at 6 weeks (Fig. 7).

### Expression of ubiquitin‐proteasome‐ and autophagy‐lysosome‐related proteins in FDS muscle

**Figure 8:**
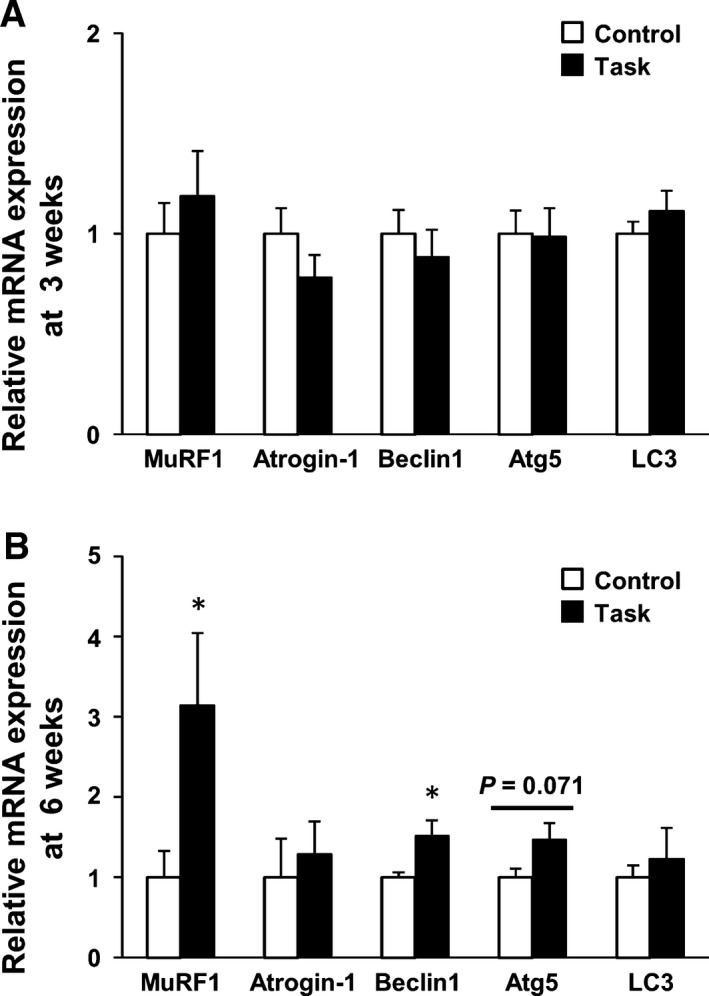
mRNA expression of E3 ubiquitin ligases and Atg genes in FDS muscles. (A) At 3 weeks. (B) At 6 weeks. Results are reported as fold changes with respect to control levels, which were arbitrarily set to 1. Data are means ± SEM. *P <* *0.05, compared with time‐matched rats in Control group (*n* = 6 per group). MuRF1, muscle RING finger 1.

MuRF1 and Atrogin‐1 were identified as two muscle‐specific E3 ubiquitin ligases, part of the ubiquitin‐proteasome pathway, that were increased transcriptionally in skeletal muscle under atrophy‐inducing conditions. Beclin1 mediates the accumulation of Atg proteins, such as the Atg5–Atg12 conjugation and LC3 conjugation systems, located in preautophagosomal structures. Such factors are considered essential for formation of the autophagic vesicle and for triggering autophagy and phagophore formation. At 3 weeks, expression of E3 ubiquitin ligases and Atg genes was not significantly different in the Control and Task groups (Fig. 8A). At 6 weeks, the Task group had significantly higher mRNA levels of MuRF1 and Beclin1, compared with the Control group (*P *<* *0.05; Fig. 8B). The Task group also had higher levels of Atg5 mRNA (*P *=* *0.071). Based on these qRT‐PCR results, we examined protein levels, at 6 weeks, of only those molecules showing differences in mRNA expression in the two groups.

**Figure 9:**
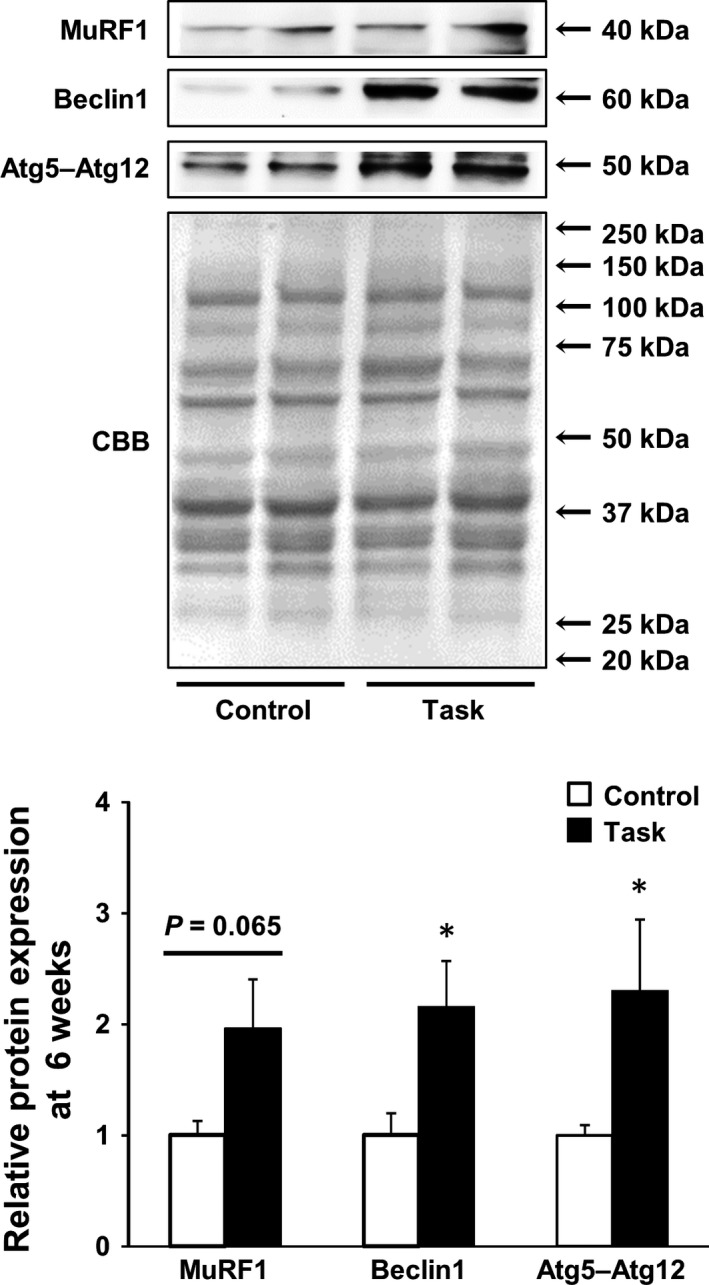
Levels of MuRF1, Beclin1, and Atg 5–Atg12 proteins in FDS muscles at 6 weeks. Representative blots depicting MuRF1, Beclin1, and Atg5–Atg12 are shown. Quantitative analysis is shown in the lower panel. Results are reported as fold changes with respect to control levels, which were arbitrarily set to 1. Data are means ± SEM. *P <* *0.05, compared with time‐matched rats in Control group (*n* = 6 per group).

By western blotting, rats in the Task group had higher levels of MuRF1 protein than those in the Control group at 6 weeks (*P *=* *0.065; Fig. 9). The Task group also had significant overexpression of the Atg proteins Beclin1 and Atg5–Atg12, relative to the Control group, at 6 weeks (both *P *<* *0.05).

## Discussion

### Change in grip strength across 6 weeks

It was reported that continuing highly repetitive (at least once every 15 s) reaching and grasping tasks for 2 h·day^−1^, 3 days per week, decreased forearm grip strength in rats, beginning at 6 weeks 5, 43. These previous studies were not designed to detect an effect at 3 weeks; though, Coq *et al*. did include a 1‐week timepoint. Therefore, in our experiment, we followed previously described methodology but investigated changes in grip strength in more detail, including at 2‐ and 3‐week timepoints. We observed, for the first time, that the decline in grip strength caused by the repetitive tasks occurred earlier than the previous study was able to discern.

### Muscle mechanical hyperalgesia caused by repetitive reaching and grasping tasks

We found, also for the first time, that repetitive reaching and grasping tasks caused muscle mechanical hyperalgesia in the rats beginning at 2 weeks. In humans, previous research showed that 23% of female workers in highly repetitive jobs reported muscle pain in the forearm and hand muscles 1. In rats, it was shown that performance of a high repetition (at least once every 15 s) handle‐pulling (force criterion 60 ± 5% of maximum voluntary force) task resulted in decreased withdrawal threshold values in the forepaws at 6 weeks 44. Other animal studies showed that skin pain resulted from highly repetitive tasks, with even higher force than those used in our experiment 45. Our study showed that forearm flexor withdrawal thresholds were significantly decreased from week 3 to week 6, compared with at week 0. These values were significantly lower in the Task than in the Control group from week 2 to week 6. Based on these results, we determined that a rat model of voluntary repetitive reaching and grasping tasks induced musculoskeletal disorders with forearm muscle pain.

### Factors associated with loss of grip strength

Examining the relationship between loss of grip strength and muscle pain, Beyreuther *et al*. 46 reported that intramuscular injection of TNF‐α caused muscle pain and decreased grip strength in rats. These investigators also showed that intramuscularly injected lacosamide or gabapentin, both having antihyperalgesic effects, improved grip strength in the presence of intramuscularly injected TNF‐α in rats. Thus, muscle pain was a contributing factor in the declined grip strength. In our study, declines in both grip strength and forearm flexor withdrawal in the Task group occurred from week 2 to week 6. These results indicated that one of the potential factors in the decline in grip strength caused by repetitive reaching and grasping tasks was muscle pain. Additional factors, relevant to extracellular matrix proteins and fibrosis, were implicated in repetitive reaching and grasping tasks. Abdelmagid *et al*. 7 reported an increase in collagen type I in skeletal muscle in rats performing the reaching and grasping tasks for 9 weeks. Frara *et al*. 47 observed increased matrix metalloproteinase levels in skeletal muscle of rats performing the tasks for 3 or 6 weeks. Cabrera *et al*. 48 showed increased collagen type I in skeletal muscle after repetitive reaching and grasping tasks, and attributed muscle fibrosis to the muscle weakness. In our study, the grip strength of rats in the Task group was lowest at 6 weeks. We observed no morphological changes in FDS muscle at 3 weeks in the Task group rats, compared with the Control group. However, relative muscle weight and muscle fiber CSA of FDS muscle were significantly decreased in Task group rats at 6 weeks. Previous studies indicated a strong correlation between muscle force and muscle volume or CSA 23, 24, 25. Thus, our findings suggested that the loss of grip strength in the Task group at 6 weeks may have been caused not only by muscle mechanical hyperalgesia, but also by muscle atrophy.

### Molecular mechanisms underlying FDS muscle atrophy

The balance between protein synthesis and degradation determines whether a muscle undergoes hypertrophy or atrophy. It was previously demonstrated that chronic low‐frequency stimulation (10 Hz, 10 h·day^−1^) decreased rat extensor digitorum longus (EDL) muscle weight after 4 weeks 49. Katzeff *et al*. 50 reported, using rats, that long‐term (4 weeks) voluntary wheel running decreased protein synthesis rates of the gastrocnemius, but not the soleus, muscle. Cunha *et al*. reported that a single session of running exercise, until exhaustion, in mice activated the 26S proteasome, leading to protein degradation in the plantaris muscle immediately after exercise. This was no longer activated at 48 h after the exercise session 32. Moreover, these investigators reported that the effects of long‐term (8 weeks) running exercise in mice, also activation of the 26S proteasome in plantaris muscle, persisted until even 48 h after the last exercise. Hence, in fast‐twitch muscles, long‐term repetitive low‐intensity muscle contraction may cause skeletal muscle atrophy by decreasing protein synthesis and increasing protein degradation. FDS muscle in the rat is known to have a high content of fast‐twitch fibers 51. Overall, the FDS muscle atrophy induced by long‐term repetitive reaching and grasping tasks (i.e., long‐term repetitive muscle contraction) likely resulted from decreased protein synthesis and increased protein degradation.

Two major protein degradation pathways, the ubiquitin‐proteasome and autophagy‐lysosome systems, are activated during muscle atrophy and contribute, to varying extents, to the loss of muscle mass. In the ubiquitin‐proteasome system, E3 ubiquitin ligase (MuRF1) can negatively regulate skeletal muscle mass. Long‐term (8 weeks) running exercise in mice elicited increased MuRF1 protein expression and 26S proteasome activity in plantaris muscle 32. Several studies showed that excessive autophagy activation aggravated muscle wasting 52, 53, 54, 55. Long‐term (4 weeks) voluntary wheel running in mice led to increased expression of Beclin1 and LC3II proteins in plantaris muscle 33. Our results showed that long‐term (6 weeks) repetitive reaching and grasping tasks increased expression of MuRF1, Beclin1, and Atg5–Atg12 proteins, suggesting induction of the ubiquitin‐proteasome and autophagy‐lysosome systems in FDS muscle. Moreover, overexpression of MuRF1, Beclin1, and Atg5, at both mRNA and protein levels, coincided temporally with FDS muscle atrophy in the Task group at 6 weeks. Consequently, we propose that activation of the ubiquitin‐proteasome and autophagy‐lysosome systems contributed to FDS muscle atrophy in the Task group.

### Relationship between muscle mechanical hyperalgesia and NGF, GDNF, and TNF‐α levels in FDS muscle

Nerve growth factor, GDNF, and TNF‐α produced muscle mechanical hyperalgesia when injected into skeletal muscle 16, 19, 41, 42. In addition, upregulation of NGF and GDNF in EDL muscle was essential to mechanical hyperalgesia in an animal model of DOMS, caused by repeated lengthening contractions 15, 16. DOMS after intense acute swimming, leading to upregulated TNF‐α in soleus muscle, was required for mechanical hyperalgesia 11. Accordingly, we measured NGF, GDNF, and TNF‐α levels in FDS muscle to investigate the mechanism of muscle mechanical hyperalgesia caused by continuing repetitive reaching and grasping tasks. Barbe *et al*. reported that performance of voluntary repetitive reaching and grasping tasks increased TNF‐α levels in rat forearm flexor muscle at 8, but not at 6 weeks 5, 43. In our study, NGF, GDNF, and TNF‐α mRNA and protein levels in the Task group were not significantly increased at 3 or 6 weeks, times when muscle mechanical hyperalgesia occurred. The observation that TNF‐α protein levels did not change in FDS muscle before 8 weeks was consistent with previous findings 5, 43. Therefore, we concluded that muscle mechanical hyperalgesia, resulting from continuing repetitive reaching and grasping tasks from week 3 to week 6, may not have been caused by NGF, GDNF, or TNF‐α. This interpretation is consistent with those of Xin *et al*. 11, who reported that inflammation and pain in a WMSD model (albeit using a different force magnitude from that in our study, i.e., a difference in intensity in task performance) do not involve the same pathway. Elliott *et al*. 10 showed that performance of voluntary repetitive reaching and grasping tasks increased levels of substance P and its preferred receptor, neurokinin‐1, in the spinal cord dorsal horn at 6 weeks. Intense noxious stimuli induce substance P release from the central terminals of dorsal root ganglion neurons. Substance P then binds to the neurokinin 1 receptor and sensitizes dorsal horn neurons. Thus, central sensitization may have contributed to the muscle pain in rats performing the voluntary repetitive reaching and grasping tasks. Further study will be necessary, investigating the roles of not only skeletal muscle, but also the central nervous system, in muscle pain caused by repetitive tasks.

In conclusion, our study demonstrated that long‐term exposure to excessive repetitive motion caused loss of grip strength, muscle pain, and skeletal muscle atrophy. These findings indicated that muscle pain can trigger a loss of grip strength within a relatively short experimental period and that skeletal muscle atrophy and muscle pain are both involved. Furthermore, such exposures may enhance protein degradation through the ubiquitin‐proteasome and autophagy‐lysosome systems, thereby decreasing skeletal muscle mass.

## Author contributions

MF, SS, MI, and TI conceived and designed the project. MF, MI, YA, NY, and KH acquired the data. MF, SS, and MI analyzed and interpreted the data and wrote the manuscript.
